# Use of the Thermal Camera to Assess the Perfusion of the Nipple-Areola Complex and Lower Adipoglandular Flap in Post-Explantation Mastopexy

**DOI:** 10.1007/s00266-024-04403-5

**Published:** 2024-11-25

**Authors:** Alberto M. Lacouture, Marco E. Guerrero, Natalia A. Lacouture, Ricardo E. Manzur

**Affiliations:** 1 Private Practice, Plastic Surgeon, Barranquilla, Colombia; 2 Simón Bolívar University, Reina Catalina Clinic, Barranquilla, Colombia

**Keywords:** Breast reconstruction, Thermography, Perfusion, Thermal image, Breast surgery safety

## Abstract

Thermography is a valuable tool for assessing tissue temperature. In recent years, explantation surgery has increased due to the fact that, despite technological advancements, breast implants have a limited lifespan and eventually require explantation, whether for pathological, aesthetic, or personal reasons. Among the complications that can arise in breast surgery are necrosis of the nipple-areola complex (NAC) and fat necrosis of the lower dermoglandular flap, which may affect the outcomes. Considering that the blood supply and perfusion of the breast were altered during the initial surgical procedure, and the technique previously used is often unknown, it is crucial to ensure adequate vascularization of the NAC and the flap. This study demonstrates the usefulness of the thermal camera for intraoperative and postoperative monitoring of the NAC and the lower adipoglandular flap, with the goal of determining tissue perfusion and identifying hypoperfused areas for immediate management, thus providing a new safety parameter in breast surgery.

*Level of Evidence V* This journal requires that authors assign a level of evidence to each article. For a full description of these Evidence-Based Medicine ratings, please refer to the Table of Contents or the online Instructions to Authors www.springer.com/00266.

## Introduction

Skin temperature is actively regulated by metabolic processes, including heat transfer from the cellular core and local blood flow. Blood serves as the primary medium for heat transport throughout the body, establishing a strong relationship between skin temperature and cutaneous perfusion [1]. Thermography is a technique that measures body temperature by detecting infrared radiation emitted from the body’s surface. These radiations, with wavelengths between 700 and 1000 nanometers (beyond the visible light spectrum), are captured by specialized sensors and converted into thermographic images. These images display different colors corresponding to the temperature recorded, which is influenced by tissue perfusion. Therefore, it can be concluded that higher temperatures generally indicate increased blood flow, while lower temperatures suggest reduced perfusion [2].

Technological advancements have enabled the integration of thermal cameras into smartphones, making this tool accessible and versatile for healthcare professionals, even during surgical procedures (preoperative, intraoperative, and postoperative). The use of thermography does not produce ionizing radiation, does not require bulky equipment, and is cost-effective, providing real-time feedback that supports prompt intraoperative decision making.

Since the surge in breast implants in 2006, the need for implant replacement or explantation (complete removal of the implant) has increased due to various reasons, including capsular contracture, implant rupture, or patient preference based on aesthetic concerns, safety issues, or implant longevity. Breast implant explantation has become one of the top ten most common surgeries in the USA [3–6]. This increase in demand has also driven the need for reconstruction with autologous tissue in cases where no new implant is placed. Several techniques have been described to restore breast volume and maintain postoperative symmetry [7, 8].

Blood perfusion may vary, particularly in previously operated breasts, where the vascular pedicle supplying the nipple-areola complex (NAC) is often unknown. This uncertainty introduces irregular patterns of vascularization, complicating the surgical procedure. While various preoperative planning methods are available, they are not widely used due to their high cost and exposure to ionizing radiation. Thermography is being studied in reconstructive and aesthetic plastic surgery, in flap dissection based on perforator vessels and in breast reconstruction surgery; however, for explantation and mastopexy it has not been extensively explored [9].

In this study, the superior pedicle technique was employed for the NAC, along with a lower adipoglandular flap [8]. The success of both the NAC and the lower flap largely depends on adequate perfusion, which ensures tissue viability and preserves breast projection at the upper pole. This highlights the potential value of thermography in assessing blood flow in these procedures [10, 11].

The objective of this study is to demonstrate the effectiveness of a smartphone-attached thermal camera in evaluating the perfusion of the NAC and lower adipoglandular flap in 108 patients undergoing mastopexy with inverted T or L incisions, following breast explantation in Barranquilla. Serial thermographic imaging was performed preoperatively, intraoperatively, and postoperatively to assess the potential risk of necrosis by correlating temperature readings with the clinical evaluation of the NAC and lower flap. The findings suggest significant potential for the application of thermography in breast reconstruction following implant explantation.

## Materials and Methods

We conducted a prospective observational study involving 108 patients who underwent surgery in Barranquilla over a 5-year period (2018–2023), analyzing a total of 216 nipple-areola complexes (NACs) and 216 adipoglandular flaps during breast reconstruction following the explantation of breast implants for both pathological and aesthetic reasons.

*Inclusion criteria:* Women with breast implants undergoing explantation, aged 20 to 75 years.

*Exclusion criteria:* Women without breast implants, patients who had undergone previous surgeries not involving implants, and those who had undergone radiotherapy.

For thermal imaging, we used a Flir One Pro® Lt model 435-0012-03 (Teledyne) for iOS, set to IRON color mode, and attached to an iPhone® 14 Pro Max (Apple Inc. USA). Thermal imaging was performed at three stages: preoperatively, intraoperatively, and immediately postoperatively. To ensure consistency in measurements, all images were taken by the same operator, with the skin surface kept dry. Images were captured from a 30 cm distance from the area of interest, with a 15–20 second wait time prior to each image capture for calibration. The thermal camera detected areas of hypoperfusion (purple zones), necrotic areas (blue-black zones), and well-perfused areas (yellow, orange, and red zones), correlating temperature with perfusion. A series of images was analyzed to establish the relationship between color and temperature, facilitating image interpretation (Table 1). The operating room temperature was consistently maintained at 17.1 °C.

**Table 1:**
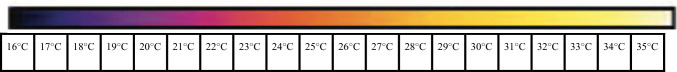
Color—temperature relationship

In the surgical technique, a superior pedicle approach was used. A modification of the traditional triangular flap was made, transforming it into a trapezoidal adipoglandular flap. The dermis of the lower flap was removed, converting it from a dermoglandular to an adipoglandular flap. Skin closure was performed with either an inverted T-shaped suture or an L-shaped closure depending on patient requirements.

**Fig. 1:**
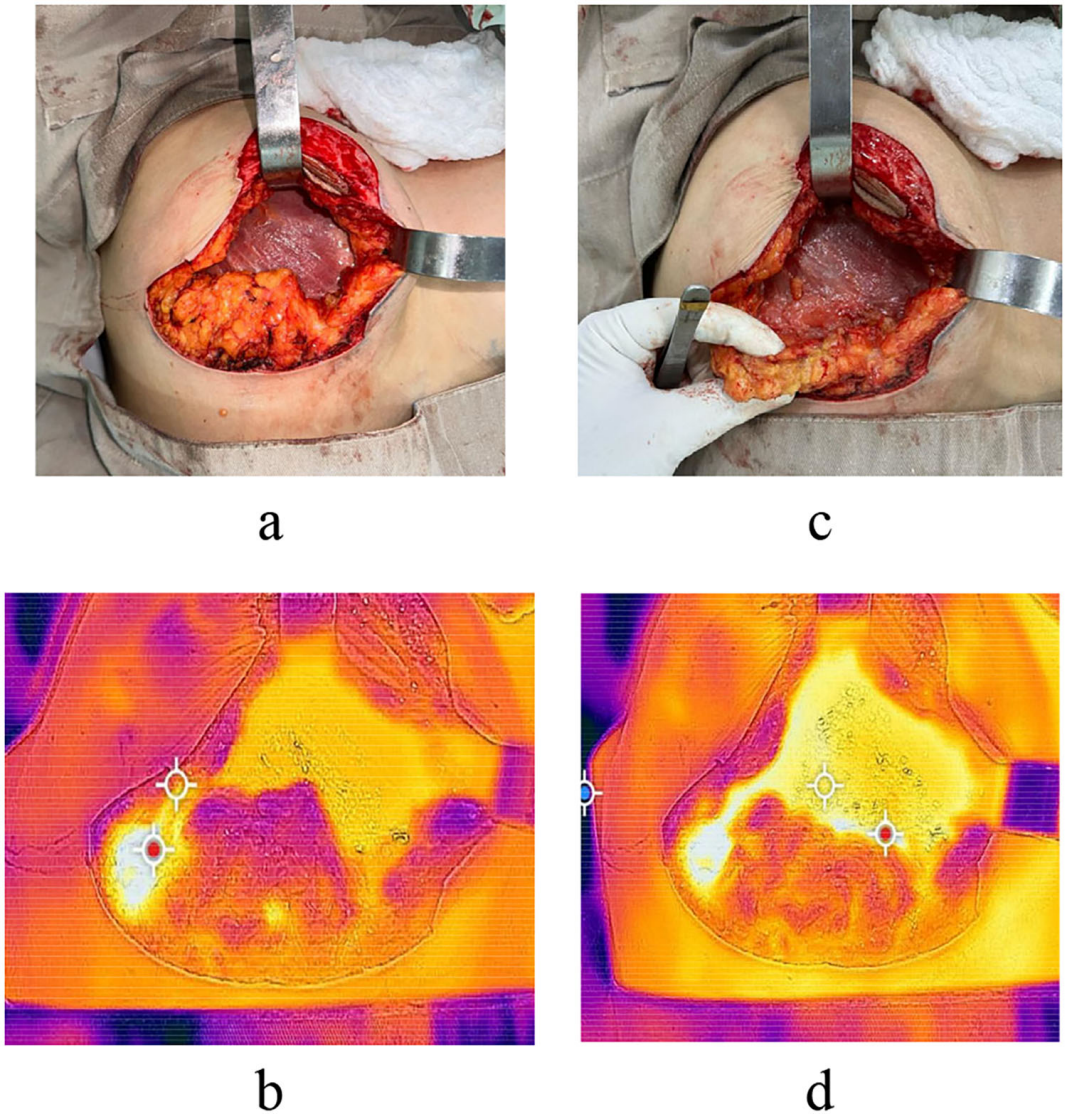
**a** Intraoperative image showing the lower flap and the nipple-areola complex (NAC) individualized. **b** Thermographic image of the flap showing hypoperfusion, with purple coloration at its distal end. **c** Image of the resection of the distal end of the lower flap. **d** Thermographic image of the lower flap with no purple coloration, indicating adequate perfusion at its distal end

During intraoperative monitoring, any hypoperfused areas of the lower flap identified as purple zones were immediately resected. A follow-up thermal image was taken to confirm the absence of these zones (Fig. 1). These images were documented along with each patient’s medical records to establish perfusion patterns and ranges.

Temperature data from the NAC and lower flap were analyzed using SPSS Statistics (IBM). All patients signed informed consent, authorizing the use of their clinical data, photographs, and images for scientific purposes.

Patients were monitored clinically and thermographically for 3 months, with weekly evaluations during the first month and monthly thereafter. Ultrasound was used to assess the lower flap for fat necrosis at both the first and third months.

## Results

Among the 108 patients, temperature variations of the NAC (nipple-areola complex) and the flap were observed across the three surgical stages. A decrease in temperature was noted from the preoperative to the intraoperative stage for both the NAC and the flap. In the postoperative stage, the temperature increased, approaching preoperative levels, while consistently maintaining values above 20 °C. We consider this the minimum allowable limit, indicating preserved perfusion. This lower threshold was established prior to the study, based on the correlation between temperature and active bleeding. Tissue registered below 20 °C did not exhibit active bleeding when tested (Table 2).

**Table 2:** Descriptive statistics distribution of NAC and flap temperature at three surgical stages

Breast		Mean	Standard deviation
Right	NAC pre	26,9	1,72
	NAC intra	26,1	1,69
	NAC post	26,4	1,66
	FLAP pre	26,3	1,85
	FLAP intra	24,7	1,61
	FLAP post	25,2	1,69
Left	NAC pre	27,2	1,84
	NAC intra	25,7	1,89
	NAC post	26,3	1,71
	Flap pre	26,6	1,95
	Flap intra	24,9	1,58
	Flap post	25,3	1,62

NAC, nipple-areola complex; Pre, preoperative; Intra, intraoperative; Post, postoperative

The temperatures recorded with the thermographic camera at various surgical stages were as follows: For the right breast in the preoperative stage, the temperature of the NAC (nipple-areola complex) was 26.9 ± 1.72 °C, and the flap was 26.3 ± 1.85 °C. Intraoperatively, the NAC temperature was 26.1 ± 1.69 °C, while the flap recorded 24.7 ± 1.61 °C. In the immediate postoperative stage, the NAC temperature was 26.4 ± 1.66 °C, and the flap was 25.2 ± 1.69 °C.

For the left breast, the preoperative NAC temperature was 27.2 ± 1.84 °C, and the flap was 26.6 ± 1.95 °C. During surgery, the NAC temperature was 25.7 ± 1.89 °C, and the flap was 24.9 ± 1.58 °C. Postoperatively, the NAC recorded 26.3 ± 1.71 °C, and the flap measured 25.3 ± 1.62 °C.

These temperature recordings were correlated with the thermographic camera’s colorimetric readings, maintaining a range from orange to yellow, as shown in Table 1. This correlation indicates a safety threshold regarding vascularization in the immediate postoperative period, with body temperatures maintained during subsequent evaluations. Colorimetric images showing temperatures below 20 °C were considered indicative of hypoperfusion and potential complications. In these cases, tissues with temperatures ≤ 20 °C were excised.

**Fig. 2:**
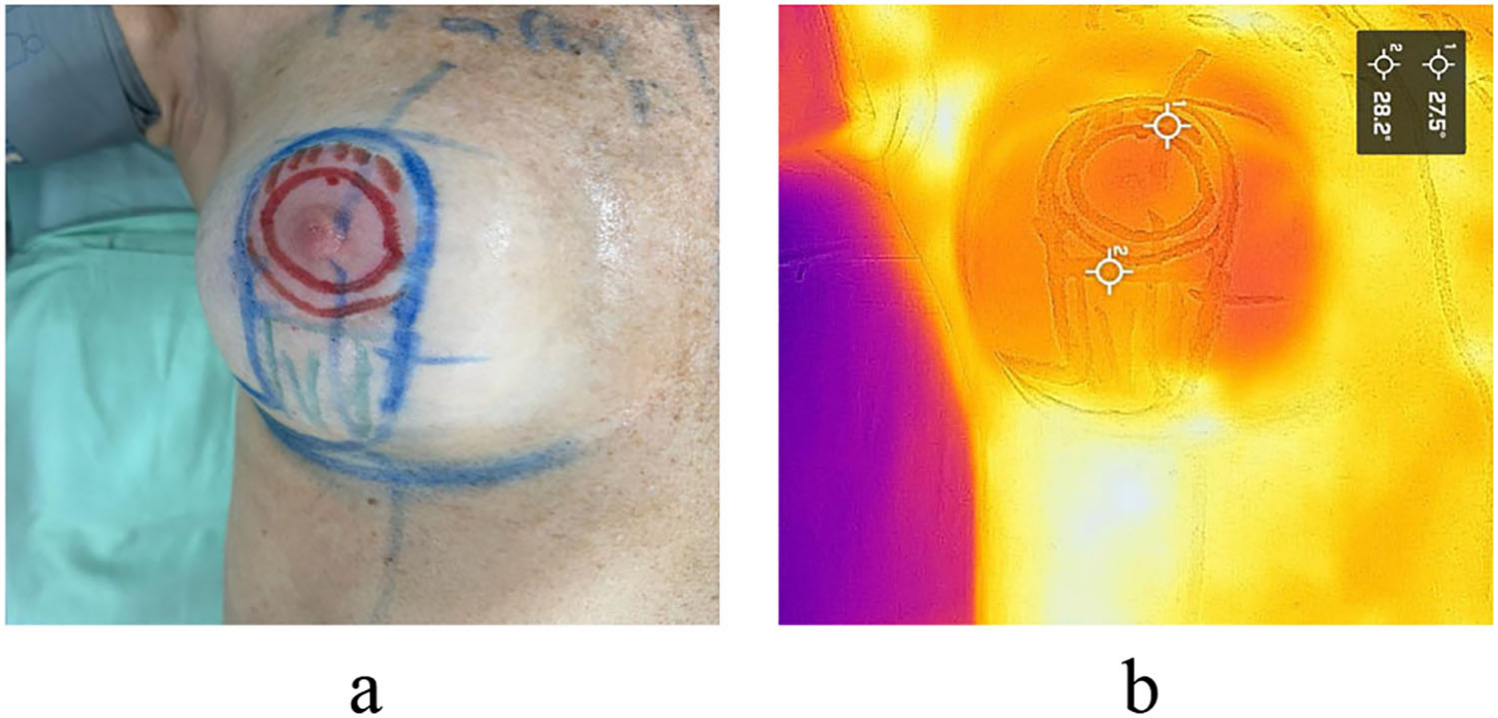
**a** Image of the right breast with markings for the superior pedicle technique. **b** Thermographic image showing the incidental finding of the breast implant’s location, characterized by lower temperature and clear delimitation

An incidental finding during the study revealed that the thermographic and preoperative colorimetric readings in the area over the breast implant differed from the rest of the chest, delineating the implant’s position beneath the skin due to lower temperatures (Fig. 2).

Regarding the lower adipoglandular flap, variations in size were observed, with widths ranging from 7 to 8 cm and lengths from 8 to 9 cm, depending on the individual case requirements. Larger flaps frequently exhibited temperatures below 20 °C at their distal edges, unlike smaller flaps. In some cases, it was necessary to excise the distal edges to maintain the appropriate width-to-length ratio of the flap.

The statistical analysis of the confidence interval and significance is presented in Table 3.

**Table 3:** Statistical analysis of confidence interval and significance in temperature variations between the NAC and lower flap

Breast		Confidence interval 95% in temperature variations		*P* value
		Inferior	Superior	
Right	NAC pre—intra	0,35	1,20	0,000
	Nac pre—post	0,04	− 0,96	0,031
	NAC intra—post	− 0,53	− 0,00	0,043
	Flap pre—intra	1,34	1,89	0,000
	Flap pre—post	0,74	1,51	0,000
	Flap intra—post	− 0,71	− 0,27	0,000
Left	NAC pre—intra	1,14	1,98	0,000
	NAC pre—post	0,50	1,43	0,000
	NAC intra—post	− 0,88	− 0,29	0,000
	Flap pre—intra	1,40	1,99	0,000
	Flap pre—post	0,86	1,61	0,000
	Flap intra—post	− 0,70	− 0,22	0,000

NAC, nipple-areola complex; Pre, preoperative; Intra, intraoperative; Post, postoperative

**Fig. 3:**
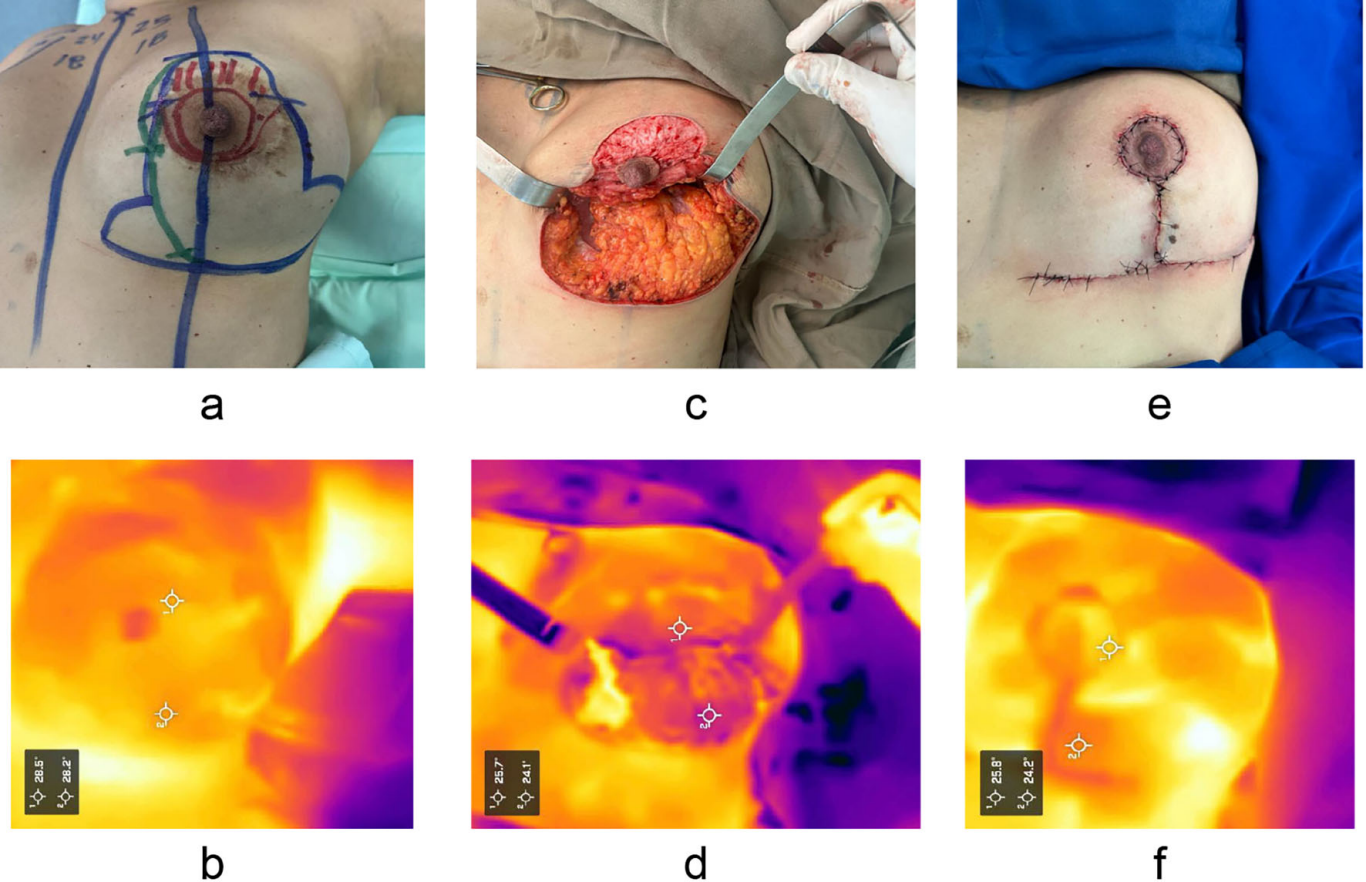
Case 1 **a** Preoperative photograph of the left breast with surgical markings: superior pedicle (red), lower adipoglandular flap (green). **b** Preoperative thermographic image. **c** Intraoperative photograph showing the NAC (nipple-areola complex) and adipoglandular flap. **d** Intraoperative thermographic image of the NAC and flap. **e** Immediate postoperative photograph with T-technique. **f** Postoperative thermographic image of the NAC and flap, showing temperatures approaching preoperative levels

**Fig. 4:**
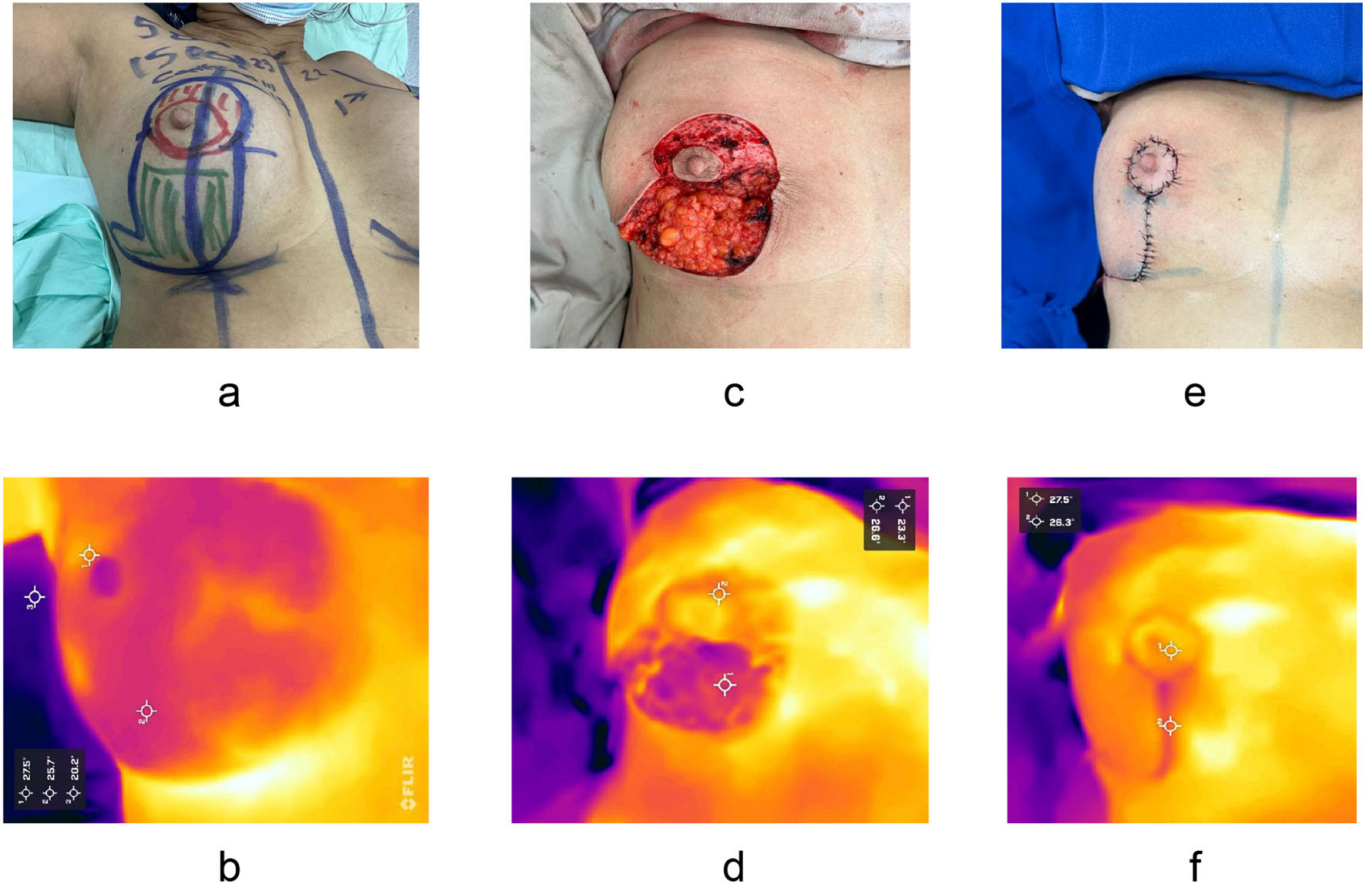
Case 2 **a** Preoperative photograph of the right breast with surgical markings: superior pedicle (red), lower flap (green). **b** Preoperative thermographic image. **c** Intraoperative photograph showing the NAC (nipple-areola complex) and lower flap. **d** Intraoperative thermographic image of the NAC and flap. **e** Immediate postoperative photograph with L-technique. **f** Postoperative thermographic image of the NAC and flap, showing temperatures approaching preoperative levels

The temperature variations recorded during the three measurement stages consistently remained above our minimum accepted threshold of 20 °C. This indicates that the temperature variation ranges are statistically significant and do not suggest any increased risk of complications for either the NAC (nipple-areola complex) or the flap (refer to Figs. 3 and 4).

## Discussion

Complications in mastopexy generally occur in approximately 10–12% of cases, with about 2% involving complications in the NAC (nipple-areola complex) and 1% related to fat necrosis of the lower flap [11]. Despite these relatively low rates, such complications can often be prevented through modifications in surgical techniques and tissue handling. To enhance safety during breast surgeries, thermographic imaging is used in conjunction with surgical techniques.

The significant variability in the location and path of blood vessels among individuals, combined with anatomical changes in breast perfusion following mammaplasty and/or breast pexy with implants, presents challenges for secondary surgeries. Thus, the thermographic camera is a valuable tool for intraoperative assessment during explantation and breast pexy using the lower adipoglandular flap, contributing to improved surgical safety [2].

Thermographic imaging has become more accessible, affordable, and user-friendly, facilitating its integration into breast surgery. Higher temperatures typically indicate increased blood flow, while lower temperatures suggest reduced blood flow [12]. This study highlights the effectiveness of thermography in predicting potential complications by providing both visual (subjective) and temperature (objective) data. Beyond these uses, thermography plays a critical role in identifying vascular pedicles for flap creation and monitoring microsurgical flaps, establishing itself as an essential tool for plastic surgeons.

## Conclusions

With advancements in technology, plastic surgeons are increasingly responsible for utilizing all available tools to ensure safe and reliable procedures for their patients. Thermography, a noninvasive and accessible technique, allows for indirect, real-time measurement of skin temperature and perfusion. Although thermography has been employed in studies of perforator flaps, its application in plastic surgery has been relatively limited. This article underscores the utility and ease of thermography for monitoring and predicting the viability of the nipple-areola complex (NAC) and the adipoglandular flap by correlating temperature and colorimetric data with perfusion. This makes it a valuable tool among current options for assessing NAC perfusion intraoperatively, enabling timely interventions before making incisions or performing pedicle debulking.

**Fig. 5:**
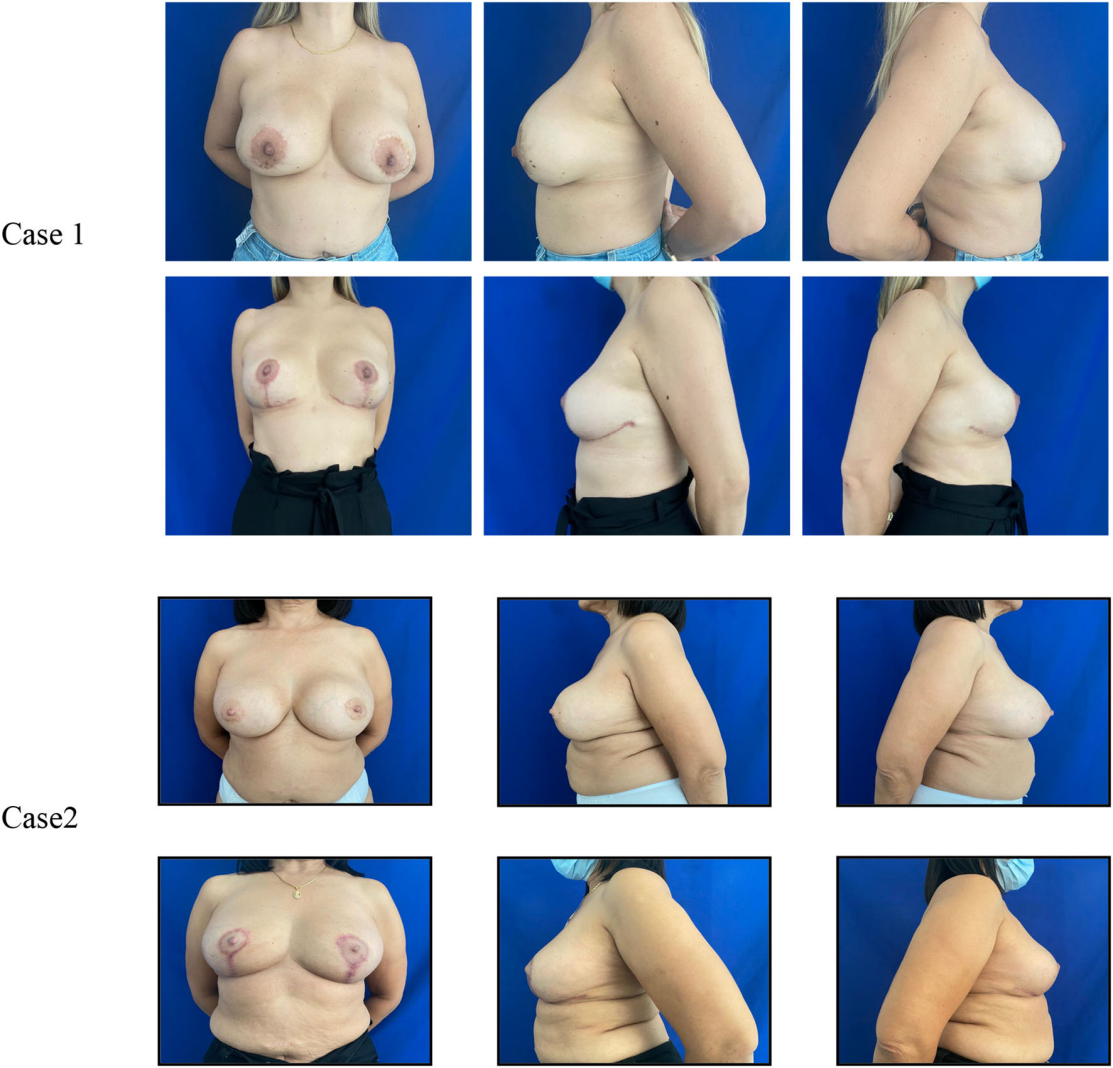
Preoperative and postoperative images of case 1 and 2 displayed in Figs. 3 and 4

In this study, no necrosis of the NAC or fat necrosis of the lower flap was observed, highlighting the potential of this technology as an adjunct for enhancing surgical safety. Thermography improves postoperative outcomes by facilitating early intervention to prevent complications such as fat necrosis of the lower flap or total/partial necrosis of the NAC (Fig. 5).

Case 1: upper row: preoperative images showing anterior, left lateral, and right lateral views of breasts with implants. Lower row: one-month postoperative images showing anterior, left lateral, and right lateral views following explantation and reconstruction with autologous tissue using the T-scar technique.

Case 2: upper row: preoperative images showing anterior, left lateral, and right lateral views of breasts with implants. Lower row: one-month postoperative images showing anterior, left lateral, and right lateral views following explantation and reconstruction with autologous tissue using the L-scar technique.
